# Pre-frailty, frailty and associated factors in older caregivers of older adults

**DOI:** 10.11606/s1518-8787.2020054001655

**Published:** 2020-01-23

**Authors:** Roberta de Oliveira Máximo, Ingrid Cristina Lopes, Allan Gustavo Brigola, Bruna Moretti Luchesi, Aline Cristina Martins Gratão, Keika Inouye, Sofia Cristina Iost Pavarini, Tiago da Silva Alexandre

**Affiliations:** I Universidade Federal de São Carlos Programa de Pós-Gradução em Fisioterapia São CarlosSP Brasil Universidade Federal de São Carlos. Programa de Pós-Gradução em Fisioterapia. São Carlos, SP, Brasil; II Universidade Federal de São Carlos Departamento de Gerontologia São CarlosSP Brasil Universidade Federal de São Carlos. Departamento de Gerontologia. São Carlos, SP, Brasil; III Universidade Federal de São Carlos Programa de Pós-Graduação em Enfermagem São CarlosSP Brasil Universidade Federal de São Carlos. Programa de Pós-Graduação em Enfermagem. São Carlos, SP, Brasil; IV Universidade Federal do Mato Grosso do Sul Três LagoasMS Brasil Universidade Federal do Mato Grosso do Sul. Curso de Medicina. Três Lagoas. MS, Brasil; V Universidade Federal de São Carlos Programa de Pós-Graduação em Gerontologia São CarlosSP Brasil Universidade Federal de São Carlos, Programa de Pós-Graduação em Gerontologia, São Carlos, SP, Brasil

**Keywords:** Caregivers, Adult, Frail Elderly, Frailty, Epidemiology, Risk Factors, Socioeconomic Factors, Cross-Sectional Studies

## Abstract

**INTRODUCTION:**

Providing care to an older adult is an activity that requires considerable physical effort and can cause stress and psychological strain, which accentuate factors that trigger the cycle of frailty, especially when the caregiver is also an older adult. However, few studies have analyzed the frailty process in older caregivers.

**OBJECTIVES:**

To investigate the prevalence of pre-frailty, frailty and associated factors in older caregivers of older adults.

**METHODS:**

A cross-sectional study was conducted including 328 community-dwelling older caregivers. Frailty was identified using frailty phenotype. Socio-demographic, behavioral and clinical aspects, characteristics related to care and functioning were covariables in the multinomial logistic regression.

**RESULTS:**

The prevalence of pre-frailty and frailty were 58.8% and 21.1%, respectively. An increased age, female sex, not having a conjugal life, depressive symptoms and pain were commonly associated with pre-frailty and frailty. Sedentary lifestyle was exclusively associated with pre-frailty, whereas living in an urban area, low income and the cognitive decline were associated with frailty. A better performance on instrumental activities of daily living reduced the chance of frailty.

**CONCLUSION:**

Many factors associated with the frailty syndrome may be related to the act of providing care, which emphasizes the importance of the development of coping strategies for this population.

## INTRODUCTION

Providing care for a dependent person is a complex task that requires substantial effort and is often only one of the many obligations of family members or informal caregivers^1^. Moreover, these individuals tend to have little technical knowledge and limited training to perform this task^2^.

Informal care is usually provided by a family member in the same age group as the care recipient (generally a wife or daughter)^3^. Besides facing their own aging process and health problems^4^, these individuals must deal with a variable and increasing load of tasks throughout the care process^5^. These tasks generally involve changes in routine, burden, stress, social isolation and the considerable expenditure of economic resources – which have physical and psychological repercussions for the caregiver^6^, making such individuals more vulnerable to diseases^8 , 9^. It is therefore plausible to suspect that the task of providing care for a dependent older adult, especially when the caregiver is also an older adult, can further increase the chance of these individuals entering the cycle of frailty.

Frailty is defined as a clinical state in which there is an increased physical and/or psychological vulnerability to the development of dependence and/or even mortality when someone is exposed to stressors^10 , 11^. The relationship between providing care and developing frailty in older caregivers of older adults has been explored in the literature. However, the association between frailty and the number of years and hours per day dedicated to providing care, financial and social support offered to the caregiver, and the burden that these individuals experience have not yet been fully clarified.

A case-control study conducted in Belgium with 79 caregivers of spouses and 79 controls found that the older caregivers had a greater chance of being frail, a greater use of antidepressants, shorter nights of sleep and greater difficulty in maintaining social contacts compared with those who were not caregivers. However, neither caregiver burden nor the time dedicated to providing care increased the chance of frailty in this group^12^.

A cross-sectional study conducted in Brazil with 148 older caregivers of older adults recruited from healthcare services investigated the occurrence of frailty in four groups based on the presence/absence of multimorbidity and high or low care-related burden. The prevalence of pre-frailty and frailty was 46% and 35.1%, respectively, and the authors found a greater chance of frailty only in the group of caregivers with multimorbidity, regardless of care-related burden^13^.

A cohort study conducted in Belgium followed up 78 caregivers of community-dwelling spouses with cognitive or functional impairment for 16 months. The authors found that most caregivers were pre-frail at baseline and so remained throughout the study, whereas one-third became frail. Moreover, the care-related burden remained stable throughout the follow-up period, but the use of medications and anxiolytics increased^14^.

Therefore, our study aims to determine the prevalence of pre-frailty and frailty in older adults who provide care for other older adults and investigate associations with socio-demographic factors, behavioral characteristics, health status, functioning and characteristics of the care process.

## METHODS

A cross-sectional study was conducted with data from community-dwelling older caregivers (age ≥ 60 years) recruited from urban and rural areas of coverage of the 18 Family Health Units (FHU) in the city of São Carlos, southeastern region of the state of São Paulo, Brazil.

The sample was selected based on a list of the total number of households within the catchment area of these 18 centers provided by employees of the primary care units, and which had at least two older people living together. Each caregiver met the following inclusion criteria: 1) aged 60 years or older, 2) registered with and residing in the area of coverage of one of the primary healthcare centers in the city and 3) providing care to a dependent older adult living in the same household. The care recipient had to be dependent on at least one basic activity of daily living (BADL) or instrumental activity of daily living (IADL)^15^, assessed using the Katz Index^16^ and Lawton and Brody Scale^17^, respectively.

**Figure 1:**
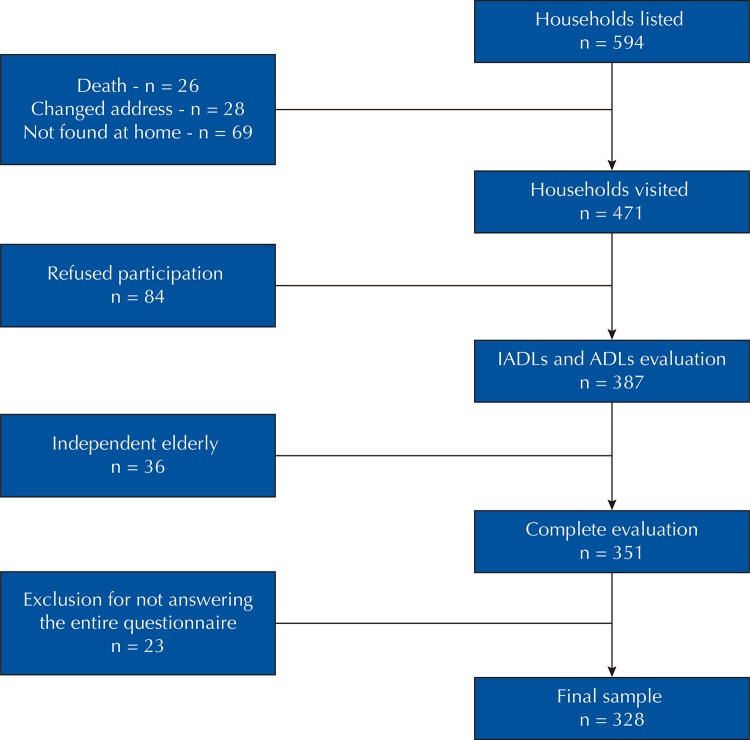
Flowchart of sample selection.

The initial number of households was 594. Of this total, 26 were excluded due to the death of one of the older adults, 28 were excluded because the residents had moved to a different address and 69 were excluded due to a lack of contact (no one at home at three attempts to visit). The remaining 471 families were visited, of which 84 were excluded because the older adults declined to participate in the study. All older adults that resided in a total of 387 households (response rate: 82.2%) were evaluated regarding functioning, and 36 families were excluded because both older adults were classified as independent considering basic and instrumental activities of daily living. Thus, older caregivers were identified and interviewed in 351 households^18^. In our study, among the 351 caregivers interviewed, 23 were excluded due to a lack of information on the variables of the frailty phenotype or the independent variables, resulting in a final sample of 328 caregivers. Figure 1 shows the flowchart of sample selection.

This project was approved by the local institutional review board (certificate number: 416.467/2013) and all participants signed an informed consent form.

### Frailty

The components of the frailty phenotype were identified using the modified version of the operational model proposed by Fried et al.^10^.

Unintentional weight loss was considered the loss of 4.5 kilograms or 5% of one’s body weight in the previous 12 months^10^.

Fatigue was analyzed using two questions based on the Center for Epidemiologic Studies Depression Scale (CES-D): a) How often have you felt that everything required considerable effort in the last week? b) How often in the past week have you felt that it is was difficult to “get going”? Fatigue was considered for older adults who reported having at least one of these feelings more than three days in the prior week^10^.

Grip strength (in kg) was measured using a dynamometer (Jamar, model SH5001), of which the highest value out of three trials was considered for the analysis. Weakness was considered for the 20% weakest individuals in each quartile of the body mass index (BMI) stratified by sex and using the cutoff points for the Brazilian population^19^. Walking speed was determined by the fastest performance out of three consecutive trials, in which every individual should walk 4.6 meters on a flat surface. Slowness was considered for the 20% slowest individuals based on mean height and stratified by sex^19^.

Physical activity level was evaluated using the Brazilian version of the International Physical Activity Questionnaire (IPAQ) and the estimate of metabolic equivalents (MET – min/week)^20^. Caloric expenditure lower than 478.15 kcal in women and 390.5 kcal in men (lowest quintile) was considered as an indicative of a low level of physical activity^19^.

Individuals with none of these components were classified as non-frail, those with one or two components as pre-frail and those with three or more components as frail^10^.

### Covariables

**Figure 2:**
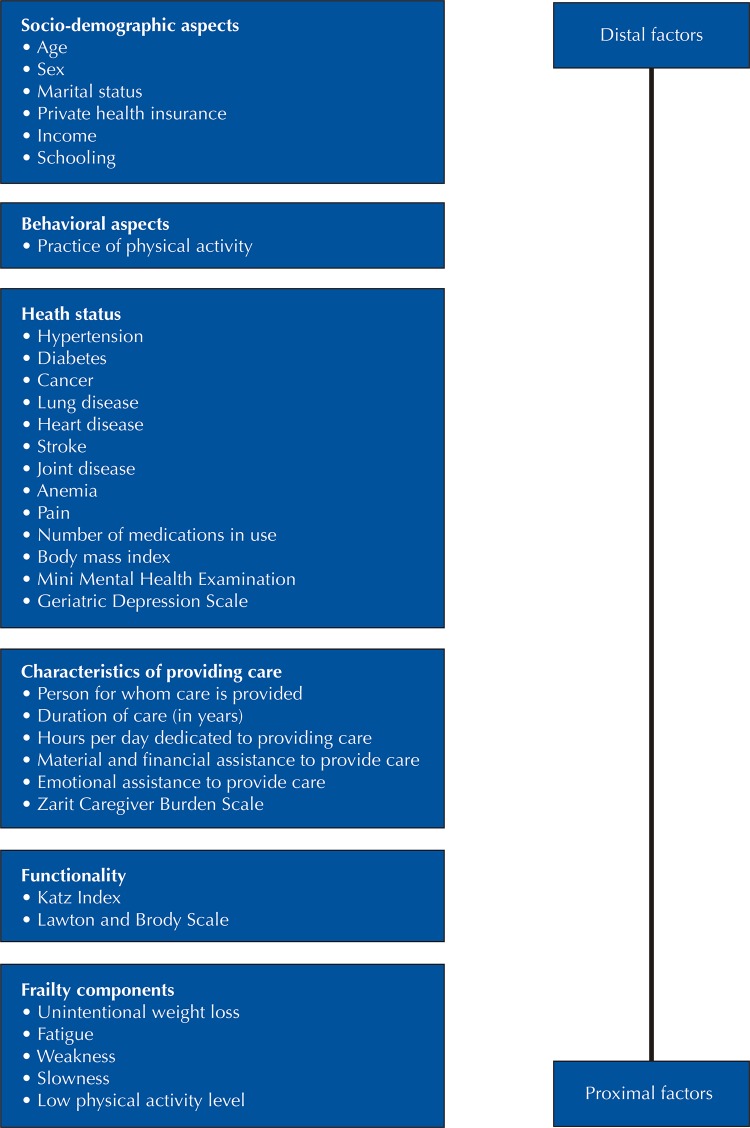
Theoretical model for investigation of factors associated with pre-frailty and frailty among older caregivers of older adults structured in hierarchical blocks.

The variables associated with pre-frailty and frailty, previously tested in a hierarchical model^19^ were included in our study along with characteristics related to providing care grouped into the following five hierarchical blocks, as shown in Figure 2:

**Socio-demographic aspects:** age and sex (caregiver and care recipient), marital status (with/without a conjugal life), private health insurance (yes/no), household income (categorized based on the Brazilian monthly minimum wage in 2014 [R$ 724.00 = US$ 222.80]), schooling level (in years) and place of residence (urban/rural);

**Behavioral aspects:** based on the results of the IPAQ, caregivers who performed less than 150 minutes of moderate or 75 minutes of vigorous physical activity per week were considered sedentary^20 , 21^;

**Health status:** self-reported arterial hypertension, diabetes, cancer, lung disease, heart disease, stroke, joint disease, anemia, pain and number of medications in use. BMI was classified according to the Pan American Health Organization recommendation^22^. The screening for cognitive decline of the caregiver and care recipient was evaluated using the Mini Mental State Examination (MMSE) with the cutoff points ≤ 19 for illiterate individuals and ≤ 23 points for those with some level of schooling^23^. Depression was analyzed using the Geriatric Depression Scale (GDS), in which a score > 5 points is considered indicative of depressive symptoms^24^;

**Care characteristics:** care recipient’s relationship to caregiver (spouse, parent, mother/father-in-law, sibling), duration of care (in years), number of hours per day dedicated to providing care, whether the caregiver receives material, financial or emotional support with regards to providing care. Caregiver burden was evaluated using the Zarit Caregiver Burden Scale. The caregivers were classified as having a low burden (0-20 points), moderate burden (21-40); moderate to severe burden (41-60) or severe burden (61-88)^25^;

**Functionality:** BADL were evaluated using the Katz Index (transferring, feeding, continence, toileting, bathing and dressing)^16^, with a score ranging between 0 and 6 points. IADL were evaluated using the Lawton and Brody Scale (using the telephone, using transportation, shopping, preparing meals, housekeeping, managing medications and managing finances)^17^, with a score ranging between 0 and 21 points. The scores of BADL and IADL were analyzed as continuous variables, with higher score indicating greater recipient’s independence.

### Statistical analysis

Simple descriptive analyses were performed to define the characteristics of the sample. The prevalence of frailty was estimated using a 95% confidence interval (CI). Factors associated with pre-frailty and frailty were analyzed using multinomial logistic regression, considering non-frail older caregivers as the reference. Associations with a *p* -value ≤ 0.20 in the univariate analyses were selected for the hierarchical modeling, which is used in epidemiological studies due to the large number of factors involved in the genesis of diseases or syndromes (making it a good method for studying frailty) and because this type of approach enables the analysis of associations on different levels. The choice of variables should be based on a theoretical model that can explain the probable pathways involved in the development of a disease^26^.

The factors analyzed were grouped into blocks and arranged according to the proximity to their influence on frailty ( Figure 2 ). The highest level included socio-demographic variables as distal factors; intermediate level included behavioral variables, and the lowest level included variables that represent health status, the characteristics of providing care and functionality. The first model was used to analyze the relationship between the frailty components and distal factors. The same strategy was adopted for the incorporation of intermediate and proximal factors in the model^19^. The identification of a statistically significant association (p < 0.05) between a particular factor and frailty showed an independent effect on the factor in question on a specific level^19^.

The Stata 14® program (Stata Corp., College Station, TX) was used for all analyses.

## RESULTS

The mean age of the care recipients was 73 years. Most recipients were men (70.7%), more than half exhibited cognitive impairment (52.8%) and the mean performance on the Katz Index was 5.1 ± 1.8 and the Lawton Scale was 13.6 ± 4.0. Among the caregivers, mean age was 69 years, 77.4% were women and the most prevalent clinical condition was systemic arterial hypertension, followed by pain, joint disease and diabetes. Regarding functionality, the caregivers showed good performance in both the Katz Index and the Lawton Scale, with averages of 5.8 ± 0.4 points e 19.2 ± 2.3 points, respectively. Regarding frailty, 58.8% (95%CI: 53.3-64.2%) of the caregivers were considered pre-frail and 21.1% (95%CI: 16.8-25.9%) were considered frail. Details of the sample characteristics are shown in Tables 1 and 2 .

**Table 1:** Descriptive socio-demographic, behavioral and health characteristics of older caregivers in São Carlos, Brazil, 2014 – 2015 (n = 328)

Socio-demographic aspects	(n = 328)
Age (years)	69.4 ± 7.1
Sex (women) (%)	77.4
Marital status (with conjugal life) (%)	89.9
Private health insurance (yes) (%)	80.5
Income	
> 5 times BMMW* (%)	11.6
2 to 5 times BMMW (%)	45.1
1 to 2 times BMMW (%)	36.3
Did not answer (%)	7.0
Schooling (years)	3.9 ± 3.5
Place of residence (urban area) (%)	76.5

**Behavioral aspects**	

Sedentary lifestyle (yes) (%)	97.3

**Health status**	

Systemic arterial hypertension (yes) (%)	64.0
Diabetes (yes) (%)	29.9
Cancer (yes) (%)	7.9
Lung disease (yes) (%)	7.9
Heart disease (yes) (%)	14.3
History of stroke (yes) (%)	2.1
Joint disease (yes) (%)	36.0
Anemia (yes) (%)	5.8
Presence of pain (yes) (%)	61.6
Body mass index	28.3 ± 5.3
Ideal range (23 < BMI < 28 kg/m^2^) (%)	35.7
Underweight (≤ 23 kg/m^2^) (%)	14.3
Overweight (28 ≤ BMI < 30 kg/m^2^) (%)	14.6
Obesity (BMI ≥ 30 kg/m^2^) (%)	35.4
Number of medications in use	3.1 ± 2.4
Mini Mental State Examination	22.9 ± 4.4
(≤ 19 – illiterate and ≤ 23 – some schooling) (%)	45.4
Geriatric Depression Scale	
(> 5 points) (%)	22.2

*Note.* Data expressed as percentage, mean and standard deviation.

BMMW = Brazilian monthly minimum wage (R$724.00 = US$222.8).

**Table 2:** Descriptive care, functionality and frailty characteristics of older caregivers in São Carlos, Brazil, 2014 – 2015 (n = 328)

Care characteristics	
Care recipient’s relationship to caregiver	
Spouse (%)	84.5
Parent (%)	7.6
Mother/father-in-law (%)	2.1
Sibling (%)	4.0
Other (%)	1.8
Duration of providing care (years)	10.0 ± 13.1
Daily hours dedicated to providing care	6.2 ± 4.8
Material and financial assistance to provide care (yes) (%)	16.8
Emotional support to provide care (yes) (%)	47.2
Zarit Caregiver Burden Scale	18.4 ± 14.5
Small burden (0 to 20) (%)	64.9
Moderate burden (21 to 40) (%)	25.0
Moderate to severe burden (41 to 60) (%)	8.9
Severe burden (61 to 88) (%)	1.2

**Functionality**	

Katz Index (points)	5.8 ± 0.4
Lawton and Brody Scale (points)	19.2 ± 2.3

**Frailty**	

Unintentional weight loss (yes) (%)	21.3
Fatigue (yes) (%)	24.4
Weakness (yes) (%)	14.9
Slowness (yes) (%)	22.9
Low physical activity level (yes) (%)	69.8
Frailty	
Non-frail (%)	20.1
Pre-frail (%)	58.8
Frail (%)	21.1

*Note.* Data expressed as percentage, mean and standard deviation.

The participants excluded were men and had a lower mean level of schooling, worse performance on the activities evaluated using the Katz Index, a greater prevalence of stroke and received less material and financial assistance when compared with the individuals included in the study (p < 0.05, data not shown).

Table 3 displays the final multinomial logistic regression model for factors associated with pre-frailty and frailty among the older caregivers of older adults. The odds ratio (OR) of the final model for factors associated with pre-frailty were 1.10 for each year of increase in age, 12.03 for caregivers without a conjugal life, 4.76 for women, 9.64 for sedentary individuals, 4.61 for those with GDS > 5 points and 2.11 for those who felt pain. The OR of the final model for factors associated with frailty were 1.17 for each year of increase in age, 15.39 for caregivers without a conjugal life, 3.59 for women, 3.85 for those residing in urban areas, 3.86 for those with income between two and five times the Brazilian monthly minimum wage, 10.21 for those with GDS > 5 points, 2.74 for those who felt pain, 2.54 for those with a MMSE score below the cutoff point adjusted for schooling and 0.70 for each unit increase on the Lawton and Brody Scale.

**Table 3:** Final multinomial logistic regression model for factors associated with pre-frailty and frailty in older caregivers of older adults in São Carlos, Brazil, 2014 – 2015

	Pre-Frail		Frail	
	
	Crude Model OR (95%CI)	Adjusted Model OR (95%CI)	Crude Model OR (95%CI)	Adjusted Model OR (95%CI)
**Model 1. Socio-demographic aspects**				

Age (in years)	1.07 (1.01 to 1.12)	**1.10 (1.04 to 1.16)**	1.14 (1.07 to 1.20)	**1.17 (1.10 to 1.24)**
Marital status				
With conjugal life	1.00	1.00	1.00	1.00
Without conjugal life	8.36 (1.10 to 63.3)	**12.03 (1.50 to 6.55)**	11.01 (1.36 to 88.68)	**15.39 (1.77 to 134.06)**
Sex				
Men	1.00	1.00	1.00	1.00
Women	3.06 (1.64 to 5.73)	**4.76 (2.34 to 9.67)**	1.86 (0.89 to 3.90)	**3.59 (1.51 to 8.58)**
Place of residence				
Rural area	1.00	1.00	1.00	1.00
Urban area	1.51 (0.82 to 2.77)	1.31 (0.67 to 2.55)	4.42 (1.74 to 11.27)	**3.85 (1.41 to 10.51)**
Income				
> 5 times BMMW*	1.00	1.00	1.00	1.00
2 to 5 times BMMW	2.07 (0.93 to 4.61)	1.77 (0.75 to 4.22)	3.38 (0.99 to 11.49)	2.20 (0.59 to 8.15)
Up to 2 times BMMW	2.84 (1.19 to 6.74)	2.25 (0.89 to 5.67)	6.79 (1.93 to 23.8)	**3.86 (1.01 to 14.74)**
Did not answer	2.10 (0.61 to 7.12)	1.52 (0.40 to 5.83)	2.10 (0.34 to 12.85)	0.94 (0.13 to 6.98)

**Model 2. Behavioral aspects**				

Level of physical activity				
Active	1.00	1.00	1.00	1.00
Sedentary	11.32 (2.29 to 55.97)	**9.64 (1.78 to 52.27)**	-	-

**Model 3. Health status**				

Geriatric Depression Scale				
≤ 5 points	1.00	1.00	1.00	1.00
> 5 points	8.90 (2.09 to 37.88)	**4.61 (1.04 to 20.53)**	23.2 (5.24 to 102.56)	**10.21 (2.16 to 48.37)**
Presence of pain				
No	1.00	1.00	1.00	1.00
Yes	1.98 (1.12 to 3.49)	**2.11 (1.08 to 4.11)**	2.58 (1.27 to 5.22)	**2.74 (1.17 to 6.42)**
Mini Mental State Examination				
No risk of cognitive impairment	1.00	1.00	1.00	1.00
Risk of cognitive impairment	2.37 (1.29 to 4.38)	1.90 (0.93 to 3.90)	3.67 (1.78 to 7.57)	**2.54 (1.06 to 6.07)**

**Model 4. Care characteristics**				

Receives emotional support				
Yes	1.00	1.00	1.00	1.00
No	1.43 (0.81 to 2.50)	1.21 (0.61 to 2.42)	1.98 (0.99 to 3.93)	1.49 (0.63 to 3.52)

**Model 5. Functional status**				

Lawton and Brody Scale	0.82 (0.69 to 0.98)	0.80 (0.62 to 1.02)	0.66 (0.55 to 0.80)	**0.70 (0.53 to 0.91)**

*Note.* OR = odds ratio. – Less than two cases. BMMW = Brazilian monthly minimum wage (R$724.00: US$222.8)

## DISCUSSION

Our main results showed that the prevalence of pre-frailty and frailty was high among older caregivers. Increased age, being woman, not having a conjugal life, depressive symptoms and pain were factors associated with both pre-frailty and frailty. A sedentary lifestyle was exclusively associated with pre-frailty, whereas living in an urban area, low income and cognitive decline were exclusively associated with frailty. Moreover, a better performance on IADL reduced the chance of frailty.

The prevalence of pre-frailty and frailty was high among the older caregivers in our study and differ from those reported in the literature. For example, in Mexican Americans, the prevalence of pre-frailty and frailty were 45.7% and 4.3%, respectively^27^. In Taiwanese individuals, Chang et al.^28^ found a 19.8% prevalence of pre-frailty and a 35% prevalence of frailty. Runzer-Colmenares et al.^29^ found a 47.3% prevalence of pre-frailty and a 27.8% prevalence of frailty in a study involving Peruvians older adults, while Moreira & Lourenço^30^ found a prevalence of 47.3% and 9.1% for pre-frailty and frailty, respectively, in the Brazilian population.

The difference in prevalence between our investigation and previous studies was expected, since our sample was composed exclusively of caregivers. This suggests that the prevalence of pre-frailty and frailty is higher among caregivers, possibly because the provision of care can easily lead to the entry into the cycle of frailty when performed by another older person, who are already more vulnerable due to their age and morbidities^31^. This hypothesis is reinforced by the findings of a study^31^ involving older Brazilian caregivers of older adults, in which the prevalence of pre-frailty (54%) and frailty (18%) are very similar to that found in our investigation.

The results of our study are consistent with earlier findings of factors associated with pre-frailty and frailty such as the increase in the prevalence of frailty with the increase in age^10 , 27 , 30 , 32 , 33^ and the greater odds of women being pre-frail or frail^10 , 32 , 33^. Women have less muscle mass and strength than men and undergo a more rapid decline in muscle strength during menopause^34^. Women also have a greater life expectancy and greater frequency of disabling chronic diseases than men^35 , 36^. Thus, women, who are generally responsible for the care provided to a spouse, mother or father, are at a biological disadvantage regarding the intrinsic physical load of providing care and have a greater chance of presenting pre-frailty and frailty when compared with men.

In the sample of our study, caregivers without a conjugal life were usually the care recipient’s son, daughter, brother or sister. Previous studies have shown that these caregivers have a worse perception regarding the reduction in or complete abandonment of professional, social and leisure activities^30^. This may imply an increase in the prevalence of depressive symptoms and exhaustion as well as a reduction in physical activity, which may explain the greater chance of pre-frailty and frailty in this group.

The occurrence of depressive symptoms was associated with both pre-frailty and frailty, which agrees with data described by Vieira et al.^32^ and Curcio et al.^37^, who found that older adults with depressive symptoms had a 160% greater chance of being frail. Depressive symptoms have been evidenced to negatively impact the homeostatic balance of the immune system^38 , 39^, increasing their chance of becoming frail. In a meta-analysis, Segerstrom and Miller^40^ found that stressful events such as providing care can cause psychosocial and physiological changes. Thus, depressive symptoms may increase the chance of older adults presenting unintentional weight loss, exhaustion and low levels of physical activity^10 , 38^, which are important components of frailty. However, we point out that the GDS and the CES-D, which are assessment tools that address exhaustion in the model proposed by Fried et al.^10^, were originally designed to measure the same phenomenon (depression) and that there could be some degree of collinearity in these associations.

Pain was associated with both frailty and pre-frailty. Persistent pain in older adults has been suggested to contribute to the frailty process through mechanisms such as reduced mobility, depression, decreased nutritional intake and the burden of comorbidities^41^. The literature shows that the physical burden of providing care may be one of the causal mechanisms of pain in older caregivers^42^.

Sedentarism was exclusively associated with pre-frailty. A sedentary lifestyle contributes to a reduction in muscle mass and strength and tolerance to exercise with ageing^10^. Moreover, taking on the burden of providing care for a family member along with heavy responsibilities other than caregiving, fatigue, anxiety and depressive symptoms may hinder the practice of physical activity^43^. Thus, the disengagement in physical activity, especially among older women with functional limitations, and the reduction in outdoor activities may explain the association between a sedentary lifestyle and pre-frailty. Such aspects have also been shown in previous studies involving older caregivers of older adults^14 , 44^.

Having a lower income and residing in an urban area were exclusively associated with frailty. Insufficient income, poverty and socio-demographic inequality can result in social and health disadvantages and cause more periods of chronic stress during the course of one’s life, affecting physical and cognitive development, especially among older caregivers^10 , 30 , 45^. Residing in an urban area agrees with data that state that must be due to the selection of mortality as well as negative impact exert by differences in lifestyle, support networks and other environmental factors. Moreover, studies have suggested that residents of rural areas are more engaged in activities, which could positively effect quality of life, thus reducing the chance of developing frailty^46^.

Cognitive impairment was associated with frailty, which agrees with findings described by Moreira & Lourenço^30^ and Curcio et al.^37^. We emphasized that Fried et al.^10^ excluded older adults with cognitive decline during the validation of the frailty phenotype, since these individuals are likely to become frail. Samper-Ternent et al.^47^ and Buchmann et al.^48^ suggest that cognitive decline and frailty syndrome may have common etiological pathways, resulting in slowness, unintentional weight loss and muscle weakness. However, further studies are needed to establish this relationship and explain the biological and psychological processes by which frailty and cognitive decline are related.

A better performance on IADL reduced the odds of being frail, which may be supported by the fact that better performance in IADL means greater participation in activities away from home, and possibly a better physical status that prevent from frailty.

Despite having analyzed factors associated with pre-frailty and frailty in older caregivers, variables related to providing care did not remain in the final model. A possible explanation may reside in the fact that the associations between frailty and an advanced age, not having a conjugal life, being a woman, having a low income, exhibiting depressive symptoms and experiencing pain per se have been strong characteristics associated with providing care in previous studies. This may have impeded associations with variables that are more directly related to the act of providing care in the present study.

Our study has some strong points. We can cite the involvement of a large sample of older caregivers of other older adults registered in primary healthcare centers. Moreover, it analyzed a broad range of variables capable of increasing the odds of frailty in older caregivers. The main limitation is the fact that a cross-sectional design does not enable the establishment of relationships of causality between the variables studied and the frailty syndrome. Also, individuals excluded due to the lack of information were less schooled, more dependent, had a higher prevalence of stroke and were predominantly men, which may have somehow prevented associations between frailty and these variables.

## CONCLUSION

The prevalence of pre-frailty and frailty was high among the older caregivers in our study. Many factors associated with the frailty syndrome may be related to the act of providing care, which emphasizes the importance of a close examination of the need for caregiver support and the development of coping strategies for this population.
